# MRI Pulse Sequences

**Published:** 1988-05

**Authors:** P. Goddard


### MRI Pulse Sequences

**P. Goddard**

The manner in which the radiofrequencies and gradients are applied are known as the pulse sequences. The particular pulse sequence chosen will determine which of the magnetic properties are most important for that particular scan. Thus by altering the sequences it is possible, for example, to weight the image in favour of the T1 magnetic property, or the T2 or proton density.

**Saturation Recovery**

The simplest sequence is known as Saturation Recovery in which there is a single 90° pulse and a long repetition time between pulses. If the signal is measured immediately following the pulse the image will depend on the proton density.

If the sequence is repeated rapidly (short repetition time, TR) there will be only partial relaxation and the results will depend on proton density and T1. Such a sequence is known as Partial Saturation Recovery.

**Inversion Recovery**

With Inversion Recovery (IR) an initial 180° pulse is used followed by a 90° pulse. The images are strongly T1 dependent. The usual time for repetition (TR) on the Picker 0.5 T scanner is around 1500 msec and time for inversion (T1) is 500 msec. Inversion Recovery sequences provide very good anatomical images.

**Spin Echo**

The third classical pulse sequence is spin echo. With Spin Echo the initial pulse is 90° and the second pulse is 180°. The results are predominantly T2 weighted but proton density (balanced) scans and T1 weighted images are provided by altering the time for repetition (TR) and time to echo (TE).

**STIR**

The STIR sequence is a variant of the Inversion Recovery in which the time to inversion (TI or Tau) is short: hence Short Tau Inversion Recovery.

The main effects of STIR are to suppress fat and summate the T1 and T2.

**MAST**

Mast is a motion artefact suppression technique used to remove the artefacts from steady or pulsatile movement such as blood or CSF flow.

**Scan Appearances**

By altering the pulse sequences and thus varying the weighting towards the various magnetic properties the signal emanating from various structures will alter remarkably. On one scan CSF may appear black whilst on another it will appear white. Fat will give a relatively high signal on nearly all scans but the STIR sequence. A simplified chart of the various blacks, greys and whites is shown below.

**Table I**

**Weighting on scans related to Sequences using 0.5 T MRI scanner**

| Sequence: | Parameters | | Weighting |
|---|---|---|---|
| Spin Echo | TR | TE | |
| | 1500 msec | 80 msec | T2 |
| | 1500 msec | 30 msec | Proton density |
| | 500 msec | 30 msec | T1 |
| Inversion Recovery | TR | TI | |
| | 1500 msec | 500 msec | T1 |
| STIR | 1500 msec | ~100 msec | Fat suppression Summation of T1 and T2 |

**Table 2**

**Simplified Chart of Scan Appearances Related to Weighting**

| Structure or Substance | *T1 weighted | *T2 Weighted | *T2 Mast | *STIR |
|---|---|---|---|---|
| Water (bulk) | Dark grey | light grey or white | light grey or white | light grey or white |
| Fat | white | light grey | light grey | dark grey or black |
| Muscle | grey | grey | grey | dark grey |
| Air | black | black | black | black |
| Cortical bone | black | black | black | black |
| Fatty bone marrow | white | light grey | light grey | black |
| Malignant tissue | dark grey | light grey or white | light grey or white | white |
| Liver | grey | grey | grey | grey or dark grey |
| Spleen | grey | grey | grey | white |
| Kidneys: Cortex and medula: ∂∂∂ | grey | light grey | light grey | white |
| Seminal vesicles | grey | light grey or white | light grey or white | white |
| Flowing blood ### | black | black | light grey or white | white |
| Brain | | | | |
| White Matter | light grey | grey | grey | grey |
| Grey matter | grey | v. light grey | v. light grey | v. light grey |
| CSF in ventricles | dark grey | light grey or white | white | light grey or white |
| CSF: "fast" flow | dark grey or black | dark grey or black | white | white |

∂∂∂ Good anatomical differentiation is seen between cortex and medulla and calyceal systems

### Depending on multi slice and flow phenomena

